# Scattering Coefficient Estimation Using Thin-Film Phantoms with a Spectral-Domain Dental OCT System

**DOI:** 10.3390/s26030815

**Published:** 2026-01-26

**Authors:** H. M. S. S. Herath, Nuwan Madusanka, Eun Seo Choi, Song Woosub, RyungKee Chang, GyuHyun Lee, Myunggi Yi, Jae Sung Ahn, Byeong-il Lee

**Affiliations:** 1 Department of Industry 4.0 Convergence Bionics Engineering, Pukyong National University, Busan 48513, Republic of Korea; sewmi96@pukyong.ac.kr; 2 Digital Healthcare Research Center, Pukyong National University, Busan 48513, Republic of Korea; 3 Department of Physics, Chosun University, Gwangju 61452, Republic of Korea; 4 Bio-Health Photonics Research Center, Korea Photonics Technology Institute, Gwangju 61007, Republic of Korea; 5 WINUS Technology Co., Ltd., Gunpo-si 15809, Republic of Korea; 6 Division of Smart Healthcare, College of Information Technology and Convergence, Pukyong National University, Busan 48513, Republic of Korea; 7 Smart Gym-Based Translational Research Center for Active Senior’s Healthcare, Pukyong National University, Busan 48513, Republic of Korea

**Keywords:** attenuation coefficient, dental imaging, OCT, scattering coefficient

## Abstract

This study introduces a framework for estimating the optical scattering properties of thin-film phantoms using a custom-built Spectral-Domain Dental Optical Coherence Tomography (DEN-OCT) system operating within the 780–900 nm spectral range. The purpose of this work was to assess the performance of this system. The system exhibited high depth-resolved imaging performance with an axial resolution of approximately 16.30 µm, a signal-to-noise ratio of about 32.4 dB, and a 6 dB sensitivity roll-off depth near 2 mm, yielding an effective imaging range of 2.5 mm. Thin-film phantoms with controlled optical characteristics were fabricated and analyzed using Beer–Lambert and diffusion approximation models to evaluate attenuation behavior. Samples representing different tissue analogs demonstrated distinct scattering responses: one sample showed strong scattering similar to hard tissues, while the others exhibited lower scattering and higher transmission, resembling soft-tissue properties. Spectrophotometric measurements at 840 nm supported these trends through characteristic transmittance and reflectance profiles. While homogeneous samples conformed to analytical models, the highly scattering sample deviated due to structural non-uniformity, requiring Monte Carlo simulation to accurately describe photon transport. OCT A-scan analyses fitted with exponential decay models produced attenuation coefficients consistent with spectrophotometric data, confirming the dominance of scattering over absorption. The integration of OCT imaging, optical modeling, and Monte Carlo simulation establishes a reliable methodology for quantitative scattering estimation and demonstrates the potential of the developed DEN-OCT system for advanced dental and biomedical imaging applications. The innovation of this work lies in the integration of phantom-based optical calibration, multi-model scattering analysis, and depth-resolved OCT signal modeling, providing a validated pathway for quantitative parameter extraction in dental OCT applications.

## 1. Introduction

Optical coherence tomography (OCT) is a powerful biomedical imaging modality that has advanced significantly over the past three decades. By using low-coherence interferometry, OCT provides noninvasive, cross-sectional images of biological tissues with micrometer-scale resolution and real-time acquisition capabilities [1,2,3,4]. These strengths have led to widespread use in ophthalmology, cardiology, dermatology, and dentistry [5,6,7]. OCT’s ability to achieve 1–3 mm imaging depth in scattering tissues, combined with compatibility with handheld and catheter-based probes, has made it indispensable for both clinical diagnostics and research [8,9]. While OCT has been traditionally dominated by qualitative morphological imaging—such as visualizing retinal layers or identifying plaques—there is growing interest in developing quantitative OCT metrics that can extract intrinsic tissue properties [10,11].

A key parameter of interest is the scattering coefficient ($\mu_{s}$), which quantifies how frequently photons are scattered as light propagates through tissue. Because $\mu_{s}$ reflects microstructural attributes such as cell density, organelle size, and refractive index variations, it provides information that cannot be inferred from structural images alone. Changes in scattering behavior are strongly associated with pathological conditions: cancerous tissues often exhibit elevated scattering due to increased nuclear size and density, whereas degenerative and atrophic tissues may show decreased scattering [12,13]. In ophthalmology, OCT-derived scattering coefficients have been linked to retinal pigment epithelium alterations and photoreceptor degeneration, providing important biomarkers for early detection of diseases such as age-related macular degeneration [14,15]. Similar trends have been reported in dentistry, where changes in scattering correlate with enamel demineralization, early caries formation, and periodontal tissue degradation [16,17]. These observations underscore the clinical potential of $\mu_{s}$, as a quantitative diagnostic marker.

However, extracting reliable scattering coefficients from OCT data remains challenging. The OCT signal is influenced by a combination of intrinsic tissue properties and system-dependent effects such as beam focusing, depth sensitivity roll-off, spectrometer noise, absorption, and multiple scattering. Traditional exponential decay models often assume single scattering, which oversimplifies the complex light–tissue interactions and can lead to errors in $\mu_{s}$ estimation [18]. To ensure accuracy, quantitative OCT methods must be validated against materials with known optical properties. This requirement creates a central role for optical phantoms, which provide controlled, reproducible environments for calibration and benchmarking [19].

Conventional phantoms—such as gels or resin-based scattering media—have been widely used, but they often lack structural precision, reproducibility, and tunability. Recent advances in 3D printing have begun to address these limitations by enabling the fabrication of phantoms with customizable geometries, spatially varying optical properties, and anatomically inspired designs [20]. Additive manufacturing techniques allow the incorporation of scattering and absorbing agents at controlled concentrations, producing phantoms with improved uniformity and reproducibility. Furthermore, multi-material 3D printing has been used to create heterogeneous and multilayered structures that more closely mimic the optical complexity of biological tissues [21,22,23]. These developments have facilitated the creation of standardized, shareable phantom models that can support cross-platform OCT calibration and performance evaluation.

Among these innovations, thin-film phantoms have emerged as particularly useful tools for validating depth-dependent OCT measurements. Thin-film phantoms consist of layers with precisely controlled thickness (from micrometers to hundreds of micrometers) and well-defined scattering particle concentrations [24]. Because many biological tissues such as skin, retina, and oral tissues are inherently layered, thin-film configurations provide a realistic yet controlled model for studying how OCT signals behave at tissue interfaces. They have been used to evaluate axial contrast, quantify sensitivity roll-off, and examine how layer geometry affects attenuation-based optical property retrieval [25]. Importantly, thin-film designs minimize bulk-scattering artifacts and enhance reproducibility, making them ideal candidates for quantitative OCT validation. When fabricated using 3D printing, these phantoms combine microstructural precision with the versatility of additive manufacturing.

In parallel, methodological advances have focused on leveraging spectral-domain OCT data to improve scattering coefficient estimation. Traditional A-scan decay fitting methods often ignore wavelength-dependent scattering and are susceptible to noise [26]. Spectral-domain analysis takes advantage of the broadband nature of OCT sources to extract wavelength-resolved scattering profiles. Spectroscopic OCT techniques have demonstrated improved tissue differentiation by exploiting differences in spectral slopes, while also mitigating confounding effects such as chromatic dispersion and depth-dependent sensitivity [27,28,29]. Integrating these approaches with well-characterized thin-film phantoms provides a path toward more accurate and reproducible extraction of $\mu_{s}$.

In this study, we propose an integrated framework for estimating the scattering coefficient of 3D-printed thin-film phantoms and for assessing imaging depth using spectral-domain OCT. Our method combines phantom fabrication, controlled spectral data acquisition, and spectroscopic analysis to derive quantitative scattering measurements. By directly comparing OCT-derived $\mu_{s}$ with the known optical properties of the phantoms, we can assess the performance of this system.

Establishing a reliable methodology for scattering coefficient estimation has important implications. Quantitative OCT metrics has promise in providing early biomarkers for diseases such as cancer, macular degeneration, and periodontal disease. Meanwhile, standardized 3D-printed phantoms can support cross-system calibration, enabling consistent quantitative OCT measurements across laboratories and clinical sites. Ultimately, the integration of spectral-domain analysis with advanced phantom fabrication can help transition OCT from a qualitative imaging tool to a robust quantitative modality capable of supporting clinical decision-making and translational research.

## 2. Materials and Methods

### 2.1. Experimental Setup

**Figure 1:**
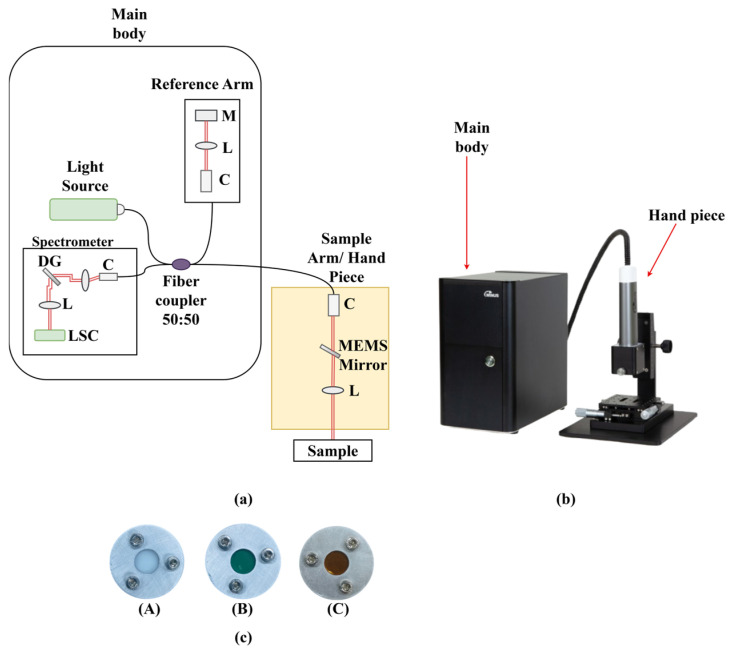
(**a**) Schematic DEN-OCT system. M: mirror; L: lens; C: collimator; DG: diffraction grating; LSC: line-scan camera; (**b**) DEN-OCT system; (**c**) thin film phantoms; (**A**) Sample A; (**B**) Sample B; (**C**) Sample C.

**Figure 2:**
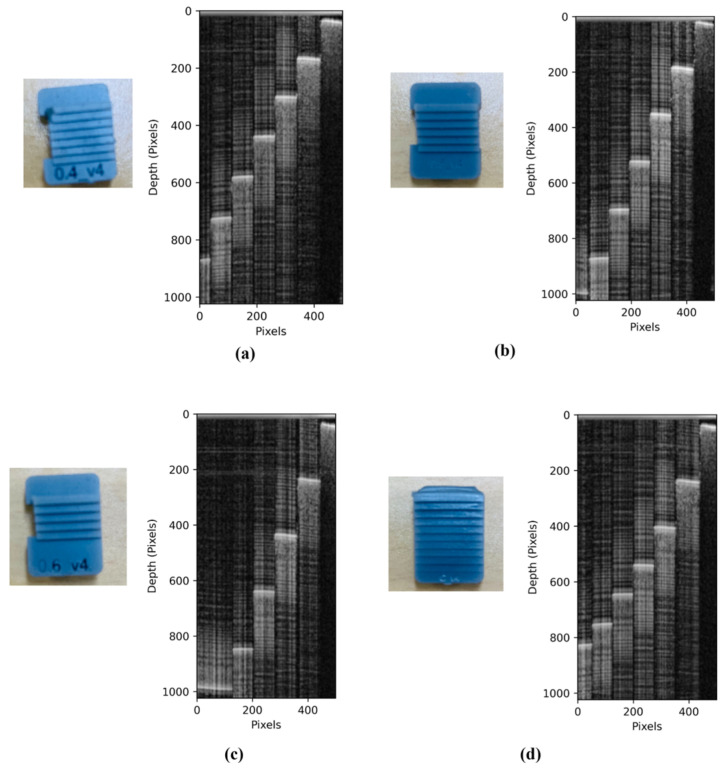
3D printed phantom and respective OCT B-scan image. (**a**) Type a phantom, (**b**) Type b phantom, (**c**) Type c phantom, (**d**) Type d phantom.

The DEN-OCT system as shown in Figure 1a and Figure 2b used in this study consists of four main components: a broadband light source, spectrometer, reference arm, and sample arm. The light source is centered at 840 nm, delivering 15 mW output power at a driving current of 180 mA, with a spectral bandwidth of 120 nm. The spectrometer (CS800–840/120, Wasatch Photonics, Morrisville, NC, USA) covers the range of 780–900 nm with a spectral resolution of 0.06 nm, providing an imaging depth of 3.0 mm. A line-scan camera is integrated into the detection path, enabling high-speed acquisition of spectral interferograms that are reconstructed into cross-sectional images. The reference arm directs part of the source light toward a mirror to generate the phase reference, with optical path length matching and dispersion compensation included to maintain signal quality.

In the sample arm, the optical beam is delivered to the target, and the backscattered light is collected for interference with the reference beam. The system supports real-time imaging and outputs images with sizes of 1000 × 1000 pixels. The lateral scan width exceeds 4.0 mm, and the overall configuration ensures stable performance and high-quality imaging across the usable depth range.

### 2.2. Measurement Settings of UV–VIS Spectrophotometer

The measurements utilized an Ocean Optics spectrophotometric system designed for high-accuracy optical analysis. A halogen light source (HL-2000-HP, Ocean Optics, Orlando, FL, USA) provided stable broadband illumination throughout the experiments. The system offered an optical angular resolution of ±0.1° FWHM, enabling precise alignment of the measurement geometry. Spectral sensing was performed using a spectrophotometer integrated with a 4-inch integrating sphere, covering a wavelength range from 380 nm to 1000 nm with a spectral resolution of 5 nm. The overall system performance ensured an accuracy of ±1% at 50% of the reference signal, providing reliable reflection, transmission, and absorption data under consistent optical conditions

### 2.3. Sample Preparation

The phantoms used in this study were designed as optical reference materials rather than full anatomical replicas of dental tissues. Their purpose is to provide controlled scattering and attenuation behavior for system calibration and methodological validation. While the materials do not replicate the complete biochemical composition of enamel or dentin, they approximate key optical regimes relevant to dental OCT, such as weakly scattering, strongly scattering, and heterogeneous media.

Thin-film tapes were obtained from Chukoh and 3M. Sample A employed polytetrafluoroethylene (PTFE) film tape ASF-110FR (Chukoh, Tokyo, Japan), Sample B used polyester film tape 8992 (3M), and Sample C consisted of polyimide film tape 5413 (3M). The single-layer thicknesses were 0.08 mm, 0.082 mm, and 0.07 mm for Samples A, B, and C, respectively. For OCT measurements, multiple layers of each tape were stacked to achieve an overall thickness of several millimeters. Fabricated phantoms labeled as A, B, and C, as shown in Figure 1c, simulate varying optical properties. Each phantom was carefully mounted within an aluminum holder to ensure stability and reproducibility during imaging. The assembly was then subjected to OCT scanning to capture high-resolution, cross-sectional images. This setup allowed for controlled evaluation of light-tissue interactions, including reflection, scattering, and attenuation within the thin film layers. The resulting OCT data provided detailed structural information and served as a basis for quantitative analysis, enabling the comparison of optical responses across the different phantom types in a standardized experimental environment.

The process begins with the preparation of the samples, which are custom-fabricated and mounted in holders for analysis. These samples are then carefully positioned under the sample arm of the OCT imaging system to ensure accurate alignment and surface focus. Once properly placed, cross-sectional images of the internal microstructure of the sample are acquired through the OCT software interface programmed using Labview displayed on the monitor. The raw images typically contain background noise, fixed-pattern artifacts, and signal contributions unrelated to the optical properties of the material, so a background removal step is performed to isolate the true sample signal. This cleaned OCT image is then converted into an intensity profile as a function of imaging depth. Because OCT penetration decreases exponentially with increasing depth due to scattering within the medium, the decay of the intensity profile carries quantitative optical information. An exponential curve is fitted to this depth-dependent decay, and from the fitted function the optical attenuation or scattering coefficient is computed. The scattering coefficient is a key parameter describing how strongly light is scattered by the material and serves as an important indicator of its internal optical structure and composition. Overall, the image provides a concise visual summary of the OCT-based analysis pipeline, beginning with physical sample preparation and ending with quantitative optical property extraction through exponential decay fitting.

### 2.4. Imaging Depth Assessment

The phantom design approach here prioritizes precise geometric structuring, aiming to provide standardized reference models for imaging depth evaluation of OCT system performance. Key design criteria included the following: (1) step heights representative of enamel thickness variations commonly observed in dental anatomy (ranging from 0.4 mm to 0.6 mm), (2) accurate assessment of imaging depth, and (3) compatibility with 3D printing to ensure manufacturing accuracy. To comprehensively evaluate system capabilities, four phantom configurations were developed. The Uniform Step Phantoms (Types a, b, and c) feature consistent step heights of 0.4 mm, 0.5 mm, and 0.6 mm, respectively. Each phantom includes six steps with a uniform width of 1.0 mm, providing reliable geometric benchmarks for resolution evaluation and step height detection. This controlled design allows for systematic comparison of how varying step sizes influence the accuracy and sensitivity of the imaging system. In contrast, the Non-Uniform Step Phantom (Type d) features a progressive decrease in step height ranging from 0.6 mm down to 0.2 mm within a single structure. This variation facilitates continuous evaluation of the system’s ability to detect geometric changes and to identify its resolution threshold.

All phantom models share the same dimensions: a total length of 12.5 mm, a width of 8 mm, and a maximum height of 3 mm, ensuring compatibility with the OCT system’s field of view and imaging depth. Each step was designed with vertical walls and minimal curvature to generate sharp optical boundaries, critical for high-precision resolution analysis. The design layout is illustrated, and the final fabricated phantoms are shown in Figure 2. These phantoms were fabricated with Grey V5 resin using Forms lab 4 SLA Printer.

Imaging depth (*ID*). Assessed to determine the maximum imaging depth achievable before signal degradation:

(1) $$ID=\sum_{i=1}^{n}h_{i}$$

where $h_{i}$ is the height of the $i^{th}$ step or layer (e.g., in a step phantom or layered sample), and *n* is the total number of steps.

The imaging depth results in Table 1 establish the upper operational depth limit of the DEN-OCT system under the given geometric conditions. This depth range defines the valid axial window within which scattering coefficients are reliably extracted from the thin-film phantoms. The consistency of the ID between 2.3 mm and 2.5 mm across different phantom materials confirms the system’s optical design stability. This finding indicates that the ID is predominantly governed by system hardware parameters such as light source wavelength and interferometer design rather than the phantom’s material composition. This stability is particularly important for standardization, as it allows for the development of reference phantoms with predictable performance regardless of the material used.

Two distinct phantom systems were intentionally employed in this study to evaluate complementary aspects of OCT performance. The geometric step phantoms were designed exclusively for system-level imaging depth and depth visibility assessment under well-defined structural conditions, independent of material scattering properties. In contrast, the thin-film phantoms (Samples A–C) were fabricated separately to enable quantitative estimation of optical scattering and attenuation coefficients. These thin-film samples were not used in the fabrication of the geometric step phantoms.

### 2.5. Calculating SNR and Roll of Curve, Axial Resolution Through the Gold-Protected Mirror

The evaluation of the OCT system using a PF10-03-M01 gold mirror (Thorlabs, Inc., Newton, NJ, USA) was carried out in three main steps: SNR and sensitivity measurement, axial resolution calculation, and depth-dependent sensitivity analysis. First, the raw spectral data, preprocessed by converting the acquired raw spectral interferogram acquired by the spectrometer, were converted to a depth-resolved A-scan. To assess SNR and roll of curve, the maximum signal was recorded using the mirror without saturating the detector, while the minimum detectable signal was measured by introducing a different length between the mirror and lens of the reference arm. The SNR was then calculated in the decibel (dB) ratio between the mean peak amplitude of the mirror signal ($A_{signal}$) and the standard deviation of the background signal ($\sigma_{noise}$) using the following formula:

(2) $$10\times \log_{10}\left(\frac{A_{Signal}}{\sigma_{noise}}\right)$$

To evaluate depth-dependent sensitivity, a sensitivity decay curve was plotted to illustrate the variation of system sensitivity with imaging depth. Finally, for the axial resolution calculation, raw spectral data were acquired by securely fixing the highly reflective and flat mirror on the sample arm and operating the OCT system to obtain A-scan signals. The full width at half maximum (FWHM) of the reflection peak in the processed A-scan was measured, ensuring that dispersion compensation was fully completed before analysis.

### 2.6. Mathematical Modeling

#### 2.6.1. Attenuation Coefficient Calculation

The attenuation coefficient ($\mu_{t}$) of a material can be determined from spectroscopic measurements of reflectance and transmittance using the Beer–Lambert law [30]. First, the reflectance R(λ) and transmittance T(λ) values are converted from percentages into fractions by dividing by 100. Since light energy is distributed among reflection, transmission, and absorption, the absorption fraction can be expressed as follows:

(3) $$A\left(\lambda \right)=1-R\left(\lambda \right)-T(\lambda )$$

To estimate the attenuation coefficient, the Beer–Lambert relation is applied to the transmittance spectrum:

(4) $$T\left(\lambda \right)=e^{-\mu_{t}d}$$

where T(λ) is the transmittance, *d* is the sample thickness, and $\mu_{t}$ is the attenuation coefficient.

Rearranging the equation gives the following:

(5) $$\mu_{t}=\;-\frac{1}{d}\ln\left(T(\lambda )\right)$$

#### 2.6.2. Reduced Scattering Coefficient Calculation

When absorption is assumed to be low compared to scattering, the transport of photons in a medium can be simplified using the diffusion approximation. In this regime, photons undergo multiple scattering events that randomize their paths, allowing their propagation to be modeled as a diffusion process rather than direct exponential attenuation described by the Beer–Lambert law [31]. The reduced scattering coefficient ($\overset{´}{\mu_{s}}$) and the attenuation of light through the medium are then governed by the effective attenuation coefficient. Experimentally, this relationship can be probed by plotting attenuation against sample thickness, where a good exponential fit confirms that the diffusion approximation is valid and allows the estimation of the effective attenuation coefficient from the slope of the fitted curve.

Here, g = 0.9 [32]:

(6) $$\mu_{eff}^{2}=3\mu_{a}(\mu_{a}+{\overset{´}{\mu }}_{s})$$

(7) $$\mathrm{with}\;{\overset{´}{\mu }}_{s}=(1-g)\mu_{s}$$

By substituting (7) to (6) we can deduce $\mu_{s}:$

$$\mu_{s\;}=\frac{\mu_{eff}^{2}-3\mu_{a}^{2}}{3\mu_{a}(1-g)}$$

### 2.7. Measure Scheme

#### 2.7.1. Sensitivity Analysis of the DEN-OCT System

The performance of the developed DEN-OCT system was quantitatively evaluated in terms of axial resolution, signal-to-noise ratio (SNR), and sensitivity roll-off characteristics. The normalized Point Spread Function (PSF) was obtained after the removal of the 0th order reflection peak. A Gaussian function was fitted to the main reflection peak to determine the system’s axial resolution. The full width at half maximum (FWHM) of the fitted curve was measured to be 16.30 µm, confirming the system’s capability for high axial resolution imaging. The primary reflection peak was located at a depth of 0.094 mm, corresponding to the reference mirror position after optical path calibration. The signal-to-noise ratio, computed as the ratio between the signal peak intensity and the average background noise level, was found to be 32.4 dB, indicating strong detection sensitivity and minimal electronic and optical noise interference.

The depth-dependent performance of the system was further analyzed by evaluating the sensitivity roll-off curve. Multiple interferometric A-scans were acquired at incremental optical path differences between the sample and reference arms to determine how sensitivity decreases with imaging depth. The measured signal amplitudes were converted to a logarithmic (dB) scale and fitted with an exponential decay model to characterize the roll-off behavior. The fitted curve revealed a gradual attenuation of signal intensity with depth, which is primarily attributed to the finite spectral resolution of the spectrometer and phase decorrelation across the detected bandwidth. The 6 dB sensitivity roll-off depth, at which the signal intensity drops by 6 dB from its maximum, was determined to be approximately at 1.81 mm.

**Figure 3:**
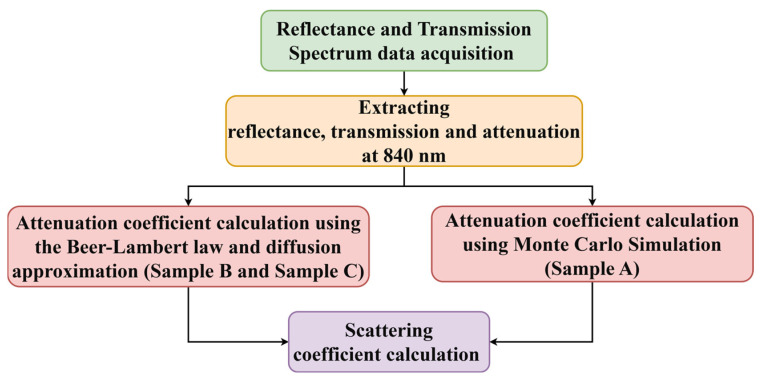
Workflow of scattering coefficient calculation.

The validation process, as illustrated in Figure 3, begins with UV–VIS spectrophotometer data acquisition, followed by the calculation of attenuation across the measured spectrum. From these data, the reflectance, transmission, and attenuation values are extracted specifically at 840 nm, which corresponds to the operating wavelength of the OCT system. Using the Beer–Lambert law, the attenuation coefficient is then determined, providing a measure of the combined absorption and scattering effects within the thin film phantom. Finally, the reduced scattering coefficient is calculated by applying the diffusion approximation model, enabling a quantitative comparison of optical scattering properties derived from spectrophotometer measurements with those obtained from OCT analysis.

Considering the results obtained, the effective imaging depth of the system was approximately 2.5 mm, beyond which the signal level becomes comparable to the noise floor. This imaging range is consistent with the theoretical limits of spectral-domain OCT systems operating in the 780–900 nm spectral window. The combined results demonstrate that the system achieves high sensitivity, stable signal decay characteristics, and a strong SNR, which together enable reliable detection of weak reflections from deeper tissue structures. These findings confirm the system’s suitability for high-resolution, depth-resolved biomedical imaging applications, such as tissue morphology analysis and optical phantom evaluation.

#### 2.7.2. Measure Scheme of the OCT A-Scan Data

The developed analytical framework provides a unified approach for estimating the optical attenuation and scattering behavior of thin-film phantoms using OCT A-scan data. The process begins by selecting a defined region of interest (ROI) in order to extract a flattened region from the OCT image, where pixel intensity profiles are extracted, smoothed, and averaged to generate representative A-scans. Peak intensities corresponding to coherent backscattered signals are then detected across the imaging depth, and their amplitudes are modeled using an exponential decay function that accounts for two-way light attenuation. By fitting the detected peaks to this model, the framework quantitatively estimates the effective attenuation coefficient ($\mu_{eff}$) and baseline intensity (C), while evaluating the goodness of fit through the coefficient of determination (R^2^). The same computational pipeline is applied consistently to all the samples A, B, and C, each characterized by distinct scattering behaviors and theoretical μ values derived from spectrophotometric or Monte Carlo analysis. This unified methodology ensures direct comparability across materials, enabling reliable assessment of scattering strength, optical uniformity, and imaging depth performance within the DEN-OCT system.

For each sample, a rectangular region of interest (ROI) was selected from the OCT B-scan in a laterally homogeneous area to avoid boundary artifacts. In the present analysis, the ROI was defined by x = 50–100 and y = 40–900 pixels (ROI size: 50 × 860 pixels). The OCT image was first denoised using a Gaussian filter (σ = 1 pixel). A depth-dependent A-scan was then obtained by averaging the ROI laterally (mean intensity across the ROI width) to suppress speckle noise and improve stability. The resulting A-scan was further smoothed using a Savitzky–Golay filter (window length = 11 samples, polynomial order = 3). Local maxima corresponding to coherent backscattered peaks were detected automatically using the find_peaks algorithm with a minimum peak spacing of 20 pixels and a prominence threshold of 0.005. Detected peaks were sorted and restricted to the valid depth range (0–2.5 mm) prior to fitting. Instead of explicit background subtraction, residual background contributions were modeled by including a constant baseline term C in the exponential decay function. Peak intensities were fitted using a two-way attenuation model in Equation (8):

(8) $$I(z)=(I_{0}-C)exp(-2\mu (z-z_{0}))+C$$

where the factor of 2 accounts for the round-trip propagation in OCT. The fitted parameters were obtained using constrained nonlinear least squares, and goodness of fit was evaluated using $R^{2}$.

## 3. Results

### 3.1. Data Analysis of UV–VIS Spectrophotometer Results

**Figure 4:**
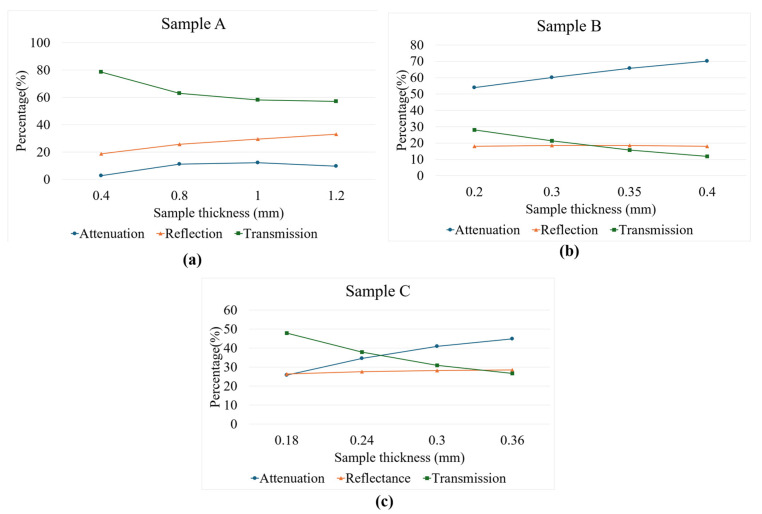
Attenuation, reflectance, and transmission for different sample thicknesses at 840 nm. (**a**) Sample A, (**b**) Sample B, (**c**) Sample C.

At 840 nm (the wavelength of the spectrometer), the three samples shown in Figure 4 clearly depict different light–matter behavior. Sample A is the most transmissive: measured transmission stays high, attenuation is low and only rises modestly with thickness, and the reduced scattering coefficient is small but increases slightly with depth. Together these indicate a material that is relatively transparent at 840 nm with scattering that becomes more significant at larger thicknesses; for OCT or other near-IR imaging this sample will permit the deepest penetration and the gentlest loss of signal with depth. Sample B behaves oppositely in that it is essentially thickness-independent, with very high attenuation, and with almost constant reflectance. This pattern indicates a strongly scattering medium in which photon loss is dominated by bulk interactions (not surface reflection), so that the light penetration at 840 nm is poor. Sample B will strongly limit the imaging depth and produce large signal loss even at thin layers. Sample C is intermediate: the reduced scattering is low and nearly constant, transmission falls markedly with thickness, while attenuation increases, and reflectance changes only slightly. This suggests a mixed regime where both scattering and absorption contribute to depth-dependent attenuation; C allows some penetration, but the signal degrades rapidly with thickness compared with A.

The optical transmittance behavior of Samples A, B, and C was analyzed using the Beer–Lambert law. This model describes an exponential relationship between light transmittance and sample thickness, providing insight into how each material attenuates light through absorption and scattering mechanisms. In all three samples, transmittance decreased with increasing thickness, confirming the general validity of the Beer–Lambert relationship.

For Sample A, transmittance gradually decreased with thickness, indicating lower optical attenuation and higher transparency compared to the other samples. The moderate correlation suggests that deviations from the Beer–Lambert law may arise from material inhomogeneity or scattering effects. Sample B showed a strong and consistent decline in transmittance, suggesting high optical density and uniform structure. This behavior reflects dominant absorption processes and minimal scattering irregularities. Sample C exhibited intermediate characteristics, with transmittance decreasing smoothly and predictably. The material demonstrates balanced absorption and scattering, with stable optical behavior within the tested range.

**Figure 5:**
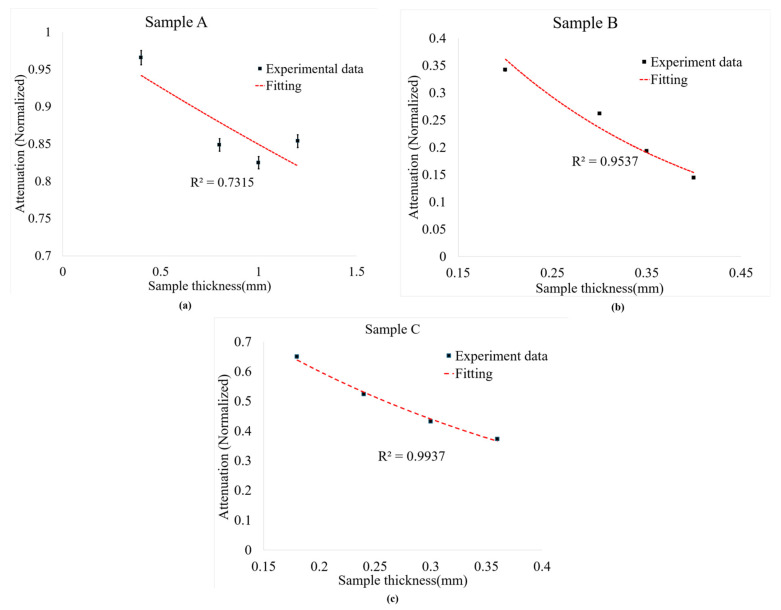
Effective attenuation coefficient calculated using the diffusion approximation across samples. (**a**) Sample A, (**b**) Sample B, (**c**) Sample C.

Figure 5 presents the diffusion approximation analysis of the same samples. This model, unlike the Beer–Lambert law, describes light propagation in turbid media dominated by multiple scattering rather than direct transmission. For Sample A, attenuation decreased only slightly with thickness and showed weak conformity to the model, likely due to internal heterogeneity and irregular scattering. Sample B, however, displayed a strong and uniform decrease in attenuation with increasing thickness, indicating highly diffusive behavior and consistent optical properties. Sample C also followed the diffusion trend closely, exhibiting moderate attenuation and uniform scattering characteristics.

Comparing both models, the Beer–Lambert law effectively explains absorption-dominated behavior, while the diffusion approximation provides a more accurate description of scattering-dominated light transport. Samples B and C demonstrated strong conformity to both models, suggesting that they possess uniform composition and predictable optical behavior. In contrast, Sample A showed weaker correlation under both conditions, indicating irregular light propagation due to scattering and non-uniformity.

Overall, both analyses confirm that light attenuation in these samples results from a combination of absorption and scattering processes. However, the diffusion approximation better represents the experimental results, particularly for Samples B and C, where multiple scattering significantly influences light transport. Therefore, the diffusion model was identified as the most suitable framework for characterizing the optical attenuation behavior of these materials.

**Figure 6:**
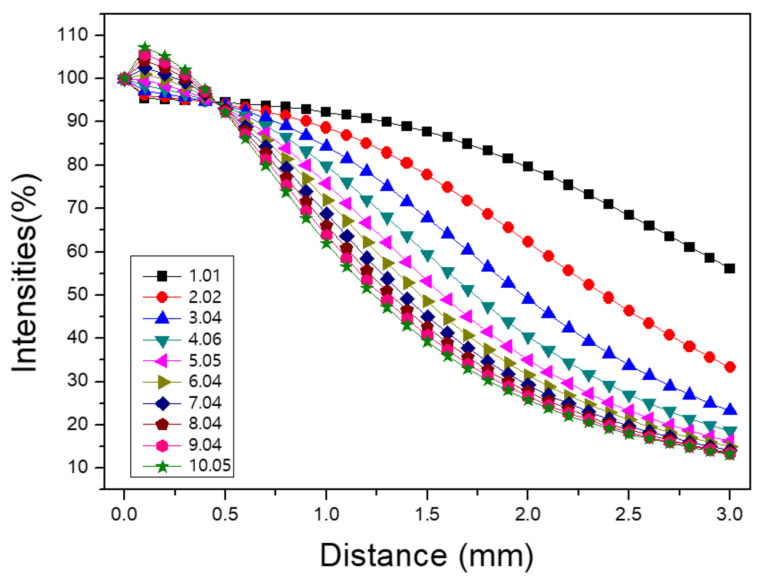
Light intensity as a percentage relative to the distance = 0.

Monte Carlo-based optical simulations were conducted using LightTools to model photon transport through scattering media. The simulation setup employed an objective lens, with a Gaussian-distributed collimated light source consisting of 10^6^ photons and an assumed beam diameter of 2.2 mm. The scattering medium was modeled with a base material refractive index of 1.6 and microspheres of 1 μm diameter having a refractive index of 1.8. The scattering coefficient ($\mu_{s}$, mm^−1^) was calculated according to Mie theory, using the microsphere concentration as a reference. It was observed that at the initial distance (distance = 0), the measured power exceeded that of the incident light due to the inclusion of reflected light components. Larger scattering particles produced higher initial reflection intensity, followed by a rapid decrease in transmitted power with depth. The simulation output includes a plot in Figure 6 of light intensity as a percentage relative to the zero point (distance = 0), where the initial intensity was normalized to 100%, demonstrating the decay characteristics of light propagation within the scattering medium. The results are summarized in Table 2.

### 3.2. Data Analysis of OCT Scan Data Results

**Figure 7:**
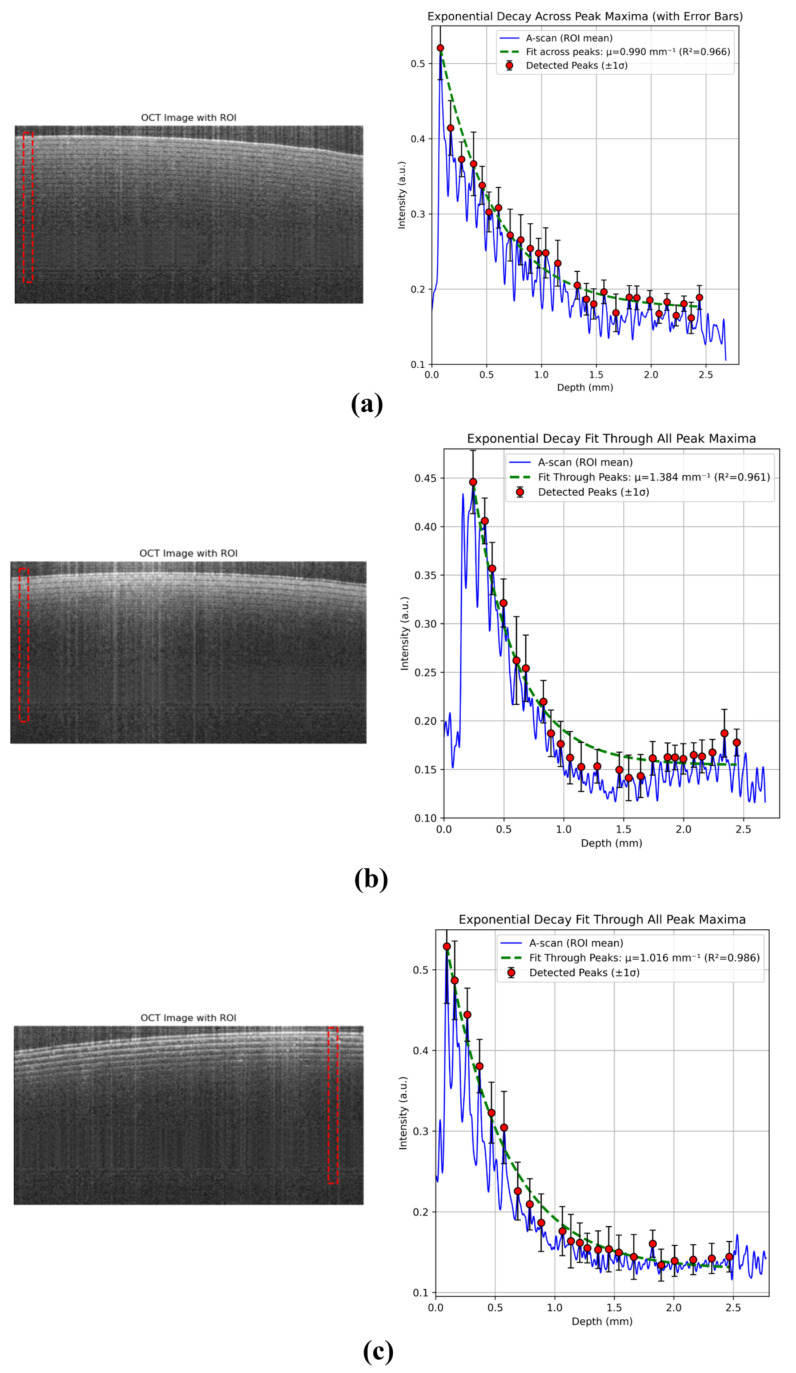
Exponential decay fit of samples (**a**) Sample A, (**b**) Sample B, (**c**) Sample C.

The OCT data of three samples are revealed in Figure 7. Table 2 shows consistent trends across the measurement methods but noticeable differences in magnitude. In particular, the UV–VIS–derived effective attenuation coefficients are generally lower than those obtained from OCT measurements. This discrepancy arises from the fundamentally different detection geometries and photon transport pathways of the two techniques. UV–VIS spectroscopy measures forward-propagating transmitted light through the entire sample thickness, yielding a bulk-averaged attenuation dominated by absorption and reduced scattering under a single-pass geometry. In contrast, OCT quantifies coherence-gated backscattered light as a function of depth, which experiences a round-trip optical path through the medium. Even under idealized single-scattering assumptions, this double-pass geometry leads to an expected increase in the apparent attenuation slope by approximately a factor of two. In practice, OCT signals are further influenced by confocal gating, depth-dependent sensitivity roll-off, and enhanced sensitivity to micro- and multiple-scattering events, which collectively bias the exponential decay toward higher effective attenuation values.

These effects are particularly evident in Table 3, where Sample B exhibits a substantial difference between Monte Carlo/UV–VIS and OCT-derived coefficients. Such a discrepancy is therefore physically reasonable and reflects the backscattering-dominated nature of OCT rather than a measurement inconsistency. From an imaging perspective, the OCT-derived coefficient more directly represents the OCT imaging performance, as it governs depth-dependent signal decay, contrast, and effective imaging depth within the same modality and detection geometry used for image formation. In contrast, UV–VIS measurements provide a complementary bulk reference of intrinsic optical behavior that is independent of OCT system characteristics.

For the homogeneous and strongly scattering samples (B and C), the OCT-to-UV–VIS ratios are relatively consistent (approximately 4–5×), suggesting that the discrepancy is systematic rather than random. This observation indicates that an empirical cross-modal scaling factor could, in principle, be introduced to map transmission-based coefficients to OCT-effective values for validation purposes, although such a factor would remain system- and geometry-dependent and was not applied in this study.

It should be noted that Sample A does not confirm to either the Beer–Lambert law or the diffusion approximation due to its weak scattering strength and structural non-uniformity, placing it in a pre-diffusive, partially ballistic transport regime. Consequently, Sample A is not suitable for direct quantitative comparison across analytical models, and Monte Carlo simulation provides a more appropriate framework for describing its photon transport behavior.

Similar magnitude discrepancies between OCT-derived attenuation coefficients and transmission-based measurements have been widely reported in OCT validation and attenuation-extraction studies, where it is well established that OCT estimates depend strongly on detection geometry, sensitivity roll-off, and backscattering dominance. The present results are therefore consistent with prior reports and underscore the importance of modality-specific interpretation of optical coefficients.

## 4. Discussion

The primary objective of this study was methodological validation rather than statistical generalization. Therefore, well-controlled phantom samples with known and repeatable optical behavior were intentionally used to demonstrate the feasibility and robustness of the quantitative extraction framework. This approach isolates system- and model-dependent effects and establishes a baseline for future biological tissue studies.

The optical characterization of the three scattering samples revealed distinct attenuation behaviors that directly influenced OCT imaging performance. The first sample exhibited the highest light transmission and the lowest surface scattering, indicating a relatively transparent medium; however, its deviation from the Beer–Lambert and diffusion models suggested internal inhomogeneity and irregular photon propagation paths. Monte Carlo simulations provided a more realistic description of this stochastic light transport, emphasizing that conventional analytical models were insufficient to fully capture their complex internal structure.

Conversely, the second and third samples demonstrated uniform and predictable attenuation characteristics that aligned closely with both analytical models, reflecting homogeneous scattering and stable internal composition. Assuming absorption effects were minimal, light attenuation in these samples was primarily governed by scattering interactions. The OCT A-scan profiles supported these findings, while the first sample showed a steep intensity decay with depth, the other two maintained stronger backscattered signals at deeper layers, indicating enhanced light transport and improved imaging depth.

Although the axial resolution and penetration depth of the DEN-OCT system are comparable to existing spectral-domain OCT systems, this does not limit its suitability for quantitative scattering extraction. Quantitative attenuation and scattering estimation primarily depend on system stability, signal-to-noise ratio, sensitivity roll-off characterization, and reproducible depth-resolved signal acquisition, rather than on achieving state-of-the-art resolution alone. In this study, system performance metrics were explicitly measured and incorporated into the analysis framework, ensuring that the extracted scattering coefficients are system-calibrated and physically meaningful.

We acknowledge that dental tissues such as enamel and dentin exhibit complex optical behavior arising from hydroxyapatite crystal orientation, tubule structure, and collagen networks, which cannot be fully described by a single scalar scattering coefficient. The present study does not aim to fully characterize dental tissue optics, but rather to establish a quantitative baseline for depth-resolved scattering estimation under controlled conditions.

The performance of the DEN-OCT system further validated its reliability for quantitative depth-resolved imaging. The system exhibited high sensitivity with a gradual roll-off in depth, stable signal-to-noise characteristics, and sufficient axial resolution for structural tissue evaluation. Minor deviations from theoretical values were likely due to experimental noise and sample surface variations. Overall, the strong correspondence among analytical modeling, Monte Carlo simulation, and OCT-based attenuation measurements confirmed the robustness of the evaluation methodology. The diffusion approximation proved suitable for homogeneous, scattering-dominant materials, whereas Monte Carlo modeling was indispensable for describing photon behavior in complex or heterogeneous media. These findings affirm the capability of the developed OCT system to accurately assess light scattering in dental and biomedical imaging applications, where quantitative interpretation of optical behavior is critical for diagnostic precision.

## 5. Conclusions

This study systematically investigated the optical behavior of three scattering samples using analytical modeling, Monte Carlo simulation, and OCT-based measurements at near-infrared wavelengths. The second and third samples exhibited uniform attenuation characteristics well-described by both the Beer–Lambert law and diffusion theory, confirming stable and homogeneous scattering properties. In contrast, the first sample displayed non-uniform behavior that required Monte Carlo simulation for accurate characterization due to its complex internal structure. OCT measurements corroborated these theoretical findings, showing a strong relationship between the optical scattering properties and the rate of signal attenuation; the depth samples with lower scattering maintained stronger OCT intensity profiles, while the non-uniform structures caused rapid signal decay and reduced penetration depth.

The comprehensive evaluation of the DEN-OCT system confirmed consistent sensitivity, adequate imaging depth, and stable signal quality, demonstrating its suitability for the optical assessment of scattering media. The integrated use of analytical modeling, simulation, and experimental OCT data provided a robust framework for characterizing light transport in turbid materials. Overall, these results highlight the potential of the developed OCT system for quantitative imaging in dental and biomedical applications, where understanding and quantifying scattering behavior are essential for improving diagnostic accuracy and imaging performance.

This work establishes a validated quantitative framework for extracting scattering-related parameters using a dental OCT system. Although demonstrated on phantom materials, the methodology provides a foundation for future tooth tissue studies, including enamel demineralization assessment, dentin structural degradation, and early caries detection. Rather than proposing a new OCT hardware platform, this study demonstrates how a clinically realistic dental OCT system can be systematically calibrated and employed for quantitative optical property extraction, supporting future translational studies on tooth tissue characterization.

## Figures and Tables

**Table 1:** ID across geometrical phantoms.

OCT Evaluation Metrics	Phantom			
	Type a	Type b	Type c	Type d
No. of visible steps	6	5	4	6
ID ^1^ (mm)	0.4 × 6 = 2.4	0.5 × 5 = 2.5	0.6 × 4 = 2.4	0.6 + 0.5 + 0.4 + 0.3 + 0.2 = 2.3

^1^ ID = Imaging Depth.

**Table 2:** Scattering coefficient results obtained by Monte Carlo method.

Concentration [Spheres/mm^3^]	Scattering Coefficient [mm^−1^]
$1.25\times 10^{6}$	1.0043
$2.5\times 10^{6}$	2.0086
$3.75\times 10^{6}$	3.0129
$5.00\times 10^{6}$	4.0170
$6.25\times 10^{6}$	5.0215
$7.5\times 10^{6}$	6.0258
$8.75\times 10^{6}$	7.0301
$1.00\times 10^{7}$	8.0344
$1.125\times 10^{7}$	9.0387
$1.25\times 10^{7}$	10.043

**Table 3:** Comparison of Monte Carlo/UV–VIS data and OCT data for samples.

Sample	Monte Carlo/UV–VIS Data [mm^−1^]			OCT Data [mm^−1^]		
	$\mu_{eff}$	$\mu_{a}$	$\mu_{s}$	$\mu_{eff}$	$\mu_{a}$	$\mu_{s}$
Sample A	-	-	1.0043	-	-	0.99
Sample B	4.274	0.16	4.144	1.384	0.16	1.224
Sample C	3.103	0.10	3.003	1.016	0.10	0.916

## Data Availability

The data presented in this study are available in the article.
